# The Corrosion Behavior of AZ91D Magnesium Alloy in Simulated Haze Aqueous Solution

**DOI:** 10.3390/ma11060970

**Published:** 2018-06-08

**Authors:** Liying Cui, Zhiyong Liu, Peng Hu, Jiamin Shao, Xiaogang Li, Cuiwei Du, Bin Jiang

**Affiliations:** 1 Corrosion and Protection Center, University of Science and Technology Beijing, Beijing 100083, China; s20161333@xs.ustb.edu.cn (L.C.); s20161372@xs.ustb.edu.cn (J.S.); lixiaogang@ustb.edu.cn (X.L.); dcw@ustb.edu.cn (C.D.); 2 Key Laboratory for Corrosion and Protection (MOE), University of Science and Technology Beijing, Beijing 100083, China; 3 Key Laboratory of Environment Fracture Ministry of Education, University of Science and Technology Beijing, Beijing 100083, China; s20161346@xs.ustb.edu.cn; 4 State Key Laboratory for Advanced Metals and Materials, University of Science and Technology Beijing, Beijing 100083, China; 5 College of Materials Science and Engineering, Chongqing University, Chongqing 400030, China; jiangbinrong@cqu.edu.cn

**Keywords:** magnesium, EIS, SEM, pitting corrosion

## Abstract

The corrosion process of AZ91D magnesium alloy in simulated haze aqueous solution has been studied by electrochemical measurements, immersion tests and morphology characterization. Results show that AZ91D was corroded heavily in simulated haze aqueous solution due to the loose and breakable product film on the surface providing little corrosion barrier. The effect of different ions was investigated. It was found that both ${NO}_{3}^{-}$ and ${NH}_{4}^{+}$ played an important role in the corrosion process. ${NO}_{3}^{-}$ helped to form passive film to protect the matrix, yet ${NH}_{4}^{+}$ consumed OH^−^, resulting in the absence of Mg(OH)_2_ and serious corrosion. Meanwhile, ${SO}_{4}^{2-}$ and Cl^−^ had influence on pitting corrosion. Magnesium aluminum oxide and MgAl_2_(SO_4_)_4_·22H_2_O instead of Mg(OH)_2_ were the dominate products, which is different from the former study. Corrosion rate changed with time, especially in the first 3 h. A two-stage corrosion mechanism is proposed after considering both the corrosion process and the influence of ions.

## 1. Introduction

Magnesium and its alloys are attractive in aerospace, automotive and electro-communication fields due to their abundant reserves and advantageous properties, such as excellent cast ability, high strength-to-weight radio and high damping capacity [1]. All these have made magnesium alloys promising metallic materials, however, the unwise use of magnesium in wet environments gives rise to its poor corrosion reputation [2]. Numerous methods, for instance, coating [3,4,5,6,7,8,9], heat treatments [10], and elements addition [11,12,13], have been used to improve the corrosion resistance of magnesium alloys. A number of studies have been undertaken to explain the corrosion behavior of magnesium. When exposed to the air, a thin and imperfect passive film will be formed on the surface. It is generally accepted that the protective film is a dense layer of MgO and Mg(OH)_2_ mixture [14,15,16], and the film is susceptible to localized breakdown. Magnesium will form a product hydroxide/oxide film when exposed to aqueous environments [17]. Santamaria et al. [18] found an ultra-thin MgO inner layer and a Mg(OH)_2_ external layer for pure magnesium after immersion in NaCl solution. Generally, the hydroxide corrosion product film on the surface of AZ91D magnesium alloy is much less stable compared with the passive film formed on the surface of aluminum and stainless steel [19]. A key step in the propagation of the corrosion is the breakage of the film [20], and tiny cracks formed on the surface expose the bare matrix to the solution to accelerate the corrosion process. Among all these corrosion types, localized corrosion, especially pitting corrosion, is the most common corrosion form of magnesium alloys. These theories are almost carried out in neutral/alkaline NaCl solution.

Nowadays, haze, as a kind of air pollution around the world, occurs when the concentration of pollutants and temperature reach a critical condition. Haze usually takes place in winter, especially January. However, pollutants exist in the air all year around. Except for the high concentration of particulate matter (PM) with an aerodynamic diameter smaller than 2.5 μm (PM2.5), the high relative humidity (80–90%) is also a significant feature of haze because the liquid membrane contains some water-soluble ions (${SO}_{4}^{2-}$, ${NO}_{3}^{-}$, ${NH}_{4}^{+}$, Cl^−^) [21] which can pose a great threat to the corrosion of magnesium. AZ91D, which is widely used nowadays, is a two-phase alloy that consists of α-matrix and β-phase, and many studies support that the β-phase can be a corrosion barrier, despite the galvanic coupling [11,22,23,24]. The corrosion behavior of metal is influenced by the environment greatly [25,26,27]. Previous studies are usually carried out in NaCl or NaSO_4_ solutions, and the corrosion performance of AZ91D in haze affected environment remains ill-defined. However, the four main kinds of water-soluble ions in haze may have an impact on the corrosion process. As a result, it is of great significance to pay attention to the corrosion behavior of AZ91D in haze circumstance in case of sudden failure in actual production and life.

The aim of this work was to study the corrosion behavior of AZ91D in simulated HA solution. The influence of immersion time and four main water-soluble ions towards corrosion was monitored by electrochemical tests, hydrogen collection, scanning electron microscopy (SEM), confocal laser scanning microscope (CLSM), energy dispersive X-ray spectroscopy (EDX) and X-ray diffraction (XRD).

## 2. Experimental Methods

### 2.1. Samples and Solution

The material studied was an as-cast AZ91D magnesium alloy. Its chemical composition is given in Table 1. Specimens with the dimension of 10 × 10 × 2 mm^3^ were prepared prior to the experiment. Samples for electrochemical measurements were mounted using epoxy resin with a 10 × 10 mm^2^ surface exposed as the working area. All exposed surface was mechanically grounded with SiC papers from 60 to 2000 grid, cleaned with acetone, distilled water and dried with cold compressed air. The microstructure of AZ91D was examined by SEM after polishing by silk polishing cloth with ethanol, washing with distilled water, drying with warm flowing air and etching in 5 vol.% nital solution.

The simulated haze aqueous solution (HA solution) used for immersion tests and electrochemical measurements was prepared according to literature [21], which suggested that the ratio of the four water-soluble ions in haze was ${SO}_{4}^{2-}$:${NO}_{3}^{-}$:${NH}_{4}^{+}$:Cl^−^ = 5:4:1:2. To stimulate HA solution and accelerate the corrosion process, the solution was set to be consisted of 0.05 M Na_2_SO_4_, 0.04 M NaNO_3_, 0.01 M NH_4_Cl and 0.01 M NaCl.

### 2.2. Electrochemical Measurements

Electrochemical measurements were performed by a VersaSTAT3 electrochemical workstation (AMETEK, Berwyn, PA, USA) with a conventional three-electrode set-up, using the samples mentioned above as working electrode, a platinum sheet as counter electrode and a saturated calomel electrode (SCE) as reference electrode. Open-circuit potential (OCP) of the specimens was monitored until it was stable with the potential changing less than 10 mV within 10 min. Electrochemical impedance spectroscopy (EIS) tests were conducted at different immersion times. The frequency ranged for EIS measurement is from 100 kHz to 10 mHz with 10 points/decade and the sinusoidal potential signal was 10 mV with respect to OCP. Potentiodynamic polarization curves were performed at a potential scanning rate of 0.5 mV/s. In this study, all potentials were measured and given with respect to SCE.

### 2.3. Morphology Observations

After immersion tests, AZ91D samples were observed by CLSM (KEYENCE, Osaka, Japan) and SEM (FEI, Hillsboro, America) equipped with EDX (AMETEK, Berwyn, PA, USA) to study the morphology and evolution of corrosion products formed on the surface. Meanwhile, the composition of the corrosion product was identified by XRD, using an automatic powder diffractometer (Rigaku, Tokyo, Japan) with monochromatic Cu Kα radiation.

A hydrogen evolution method was used to measure the corrosion rate of AZ91D in simulated HA solution [28]. The volume of the evolved hydrogen was measured by an inverted funnel with scale. Before test, the electrolyte was pre-saturated with hydrogen to reduce the error [29].

## 3. Results

### 3.1. The Corrosion Process of AZ91D

#### 3.1.1. Microstructural Characterization

**Figure 1:**
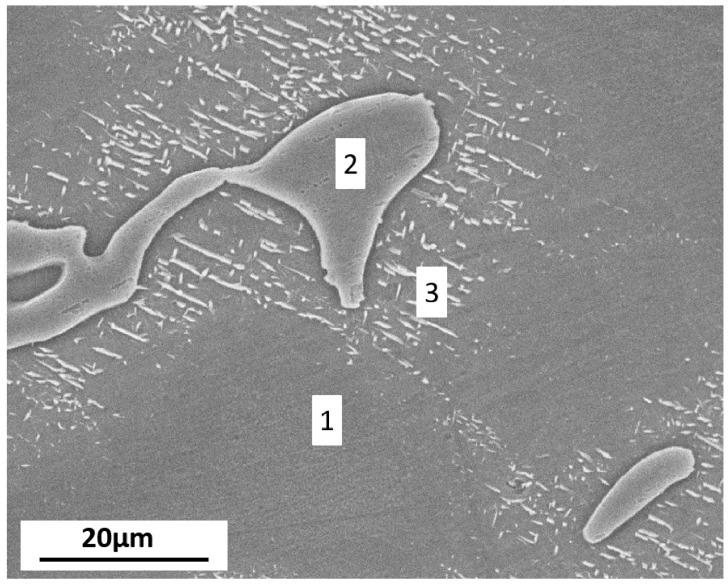
The microstructure of AZ91D.

In Figure 1, the microstructure of AZ91D alloy is presented. It shows that AZ91D is a two-phase alloy, consisting of α-matrix (marked (1) in Figure 1) and β-phase (marked (2) in Figure 1). The fine lamellar (α + β) micro-constituents are marked (3) in Figure 1, with white regions representing β-phase. The large β articles are interconnected and form a fine network throughout the microstructure. Further, the element composition of regions 1 and 2 measured by EDX is presented in Table 2.

#### 3.1.2. Electrochemical Measurements

**Figure 2:**
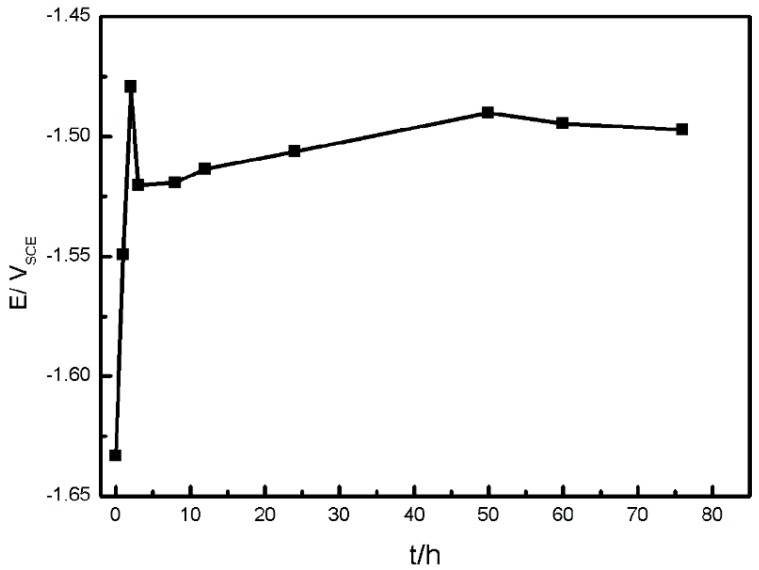
OCP dependence on immersion time of AZ91D in simulated HA solution.

The variation of OCP during the immersion time presents the chemical stability of working electrode, through which we can infer the corrosion process of samples. Figure 2 presents the OCP during immersion tests in simulated HA solution for 76 h.

As shown in Figure 2, the OCP of AZ91D is affected by immersion time, especially in the first three hours. The original potential is −1.63 V and after the sample is immersed in simulated HA solution for 3 h, the OCP approaches to −1.48 V, raised by 0.15 V, suggesting the formation of slightly protective corrosion film on the surface. However, the OCP drops suddenly after 3 h immersion, and then increases slowly as the immersion time increased, which is likely due to the breakage of protective film and the development of pitting.

**Figure 3:**
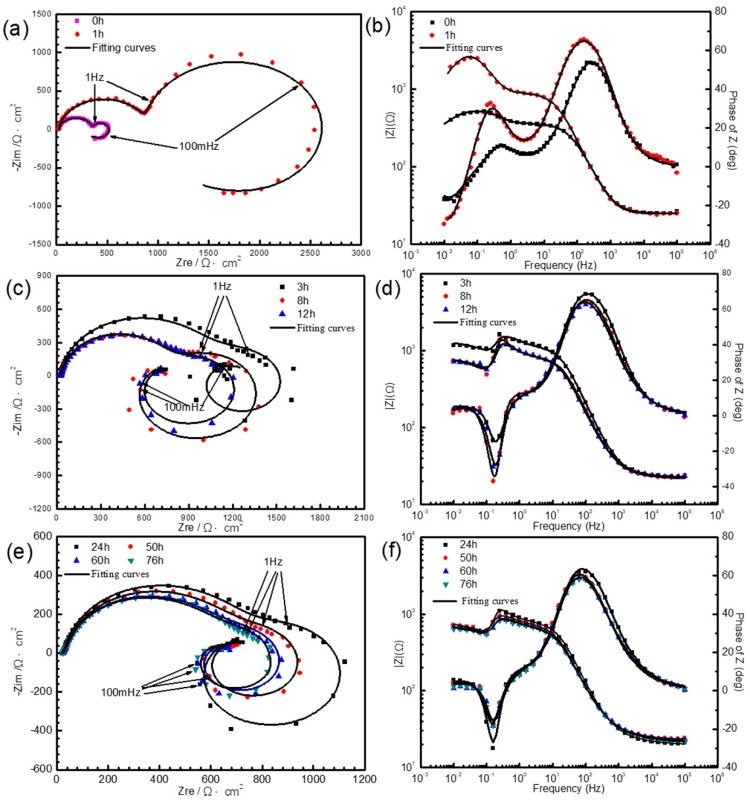
EIS dependence on immersion time of AZ91D in simulated HA solution: (**a**,**c**,**e**) Nyquist (**b**,**d**,**f**) Bode.

After immersion in simulated HA solution for various times up to 76 h, Nyquist and Bode diagrams of AZ91D sample are depicted in Figure 3, exhibiting various electrochemical characteristics.

As can be seen from Figure 3a, when the immersion time is less than 3 h, the Nyquist plots are characterized by two well-defined capacitive loops: one capacitive loop is at high frequencies and the other is at medium frequencies. In addition, there is also an inductive loop at low frequencies. Both the high frequencies and medium frequencies capacitive loop increase over the immersion time. In the meantime, the medium frequencies loop grows much faster than the high frequencies one.

As shown in Figure 3c,e, when the immersion time reaches 3 h, the fourth constant time is emerged: a new capacitive loop in the low frequencies range. Meanwhile, the medium frequencies capacitive loop decreases evidently. With the further increase of immersion time, the high frequencies and medium frequencies capacitive loop and medium frequencies inductive loop all degenerate, while the low frequencies capacitive loop changes a little.

These results are similar to those reported by Chen [30] et al. It has been suggested that the high frequencies capacitive loop is attributed to the charge transfer resistance and double capacitance at the mental/solution interface. They proposed that the capacitive loop in the medium frequencies range is induced by film effect in the corrosion process, which referred to the formation of Mg(OH)_2_ and MgAl_2_(OH)_8_·H_2_O, meanwhile the capacitive loop in the low frequencies range could be attribute to localized corrosion and the formation of MgAl_2_(SO_4_)_4_. However, in this study Mg(OH)_2_ and MgAl_2_(OH)_8_·H_2_O could not be detected due to ${NH}_{4}^{+}$, which will be described below, so we speculate that the film effect resulting in capacitive loop in the medium frequencies range may refer to the formation of MgO, one of the main products in this article. The low frequencies inductive loop is produced by the metastable Mg^+^ concentration [30,31].

**Figure 4:**
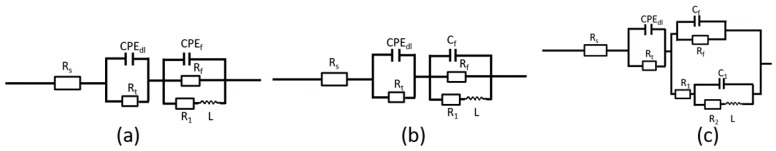
Equivalent circuits for AZ91D with different exposure time in simulated HA solution: (**a**) Model A, (**b**) Model B, (**c**) Model C.

In order to better understand the electrochemical processes of the AZ91D in the HA environment, the equivalent circuits were introduced as shown in Figure 4. When the sample was just immersed in the simulated HA solution, the equivalent circuit could be attained (Model A): a solution resistance *R*_s_, a charge transfer resistance *R*_t_, in parallel with the double layer constant phase element CPE_dl_, film resistance *R*_f_, in parallel with film constant phase element CPE_f_, and the inductance L induced by metastable Mg^+^ ions in series with a charge transfer resistance *R*_1_; When the immersion time was 1 h, the equivalent circuit could be obtained (Model B): CPE_f_ is altered to *C*_f_, indicating the film effect became relatively stable; when the immersion time was more than 3 h, the equivalent circuit could also be obtained (Model C): *C*_1_ is assigned to the layer where localized corrosion occurred, and *R*_2_ is the MgAl_2_(SO_4_)_4_ film resistance, and the other parameters are consistent with those in Model B. It can be seen from Figure 3 that the equivalent circuits fit the experimental data well, implying that equivalent circuits in Figure 4 are suitable.

**Figure 5:**
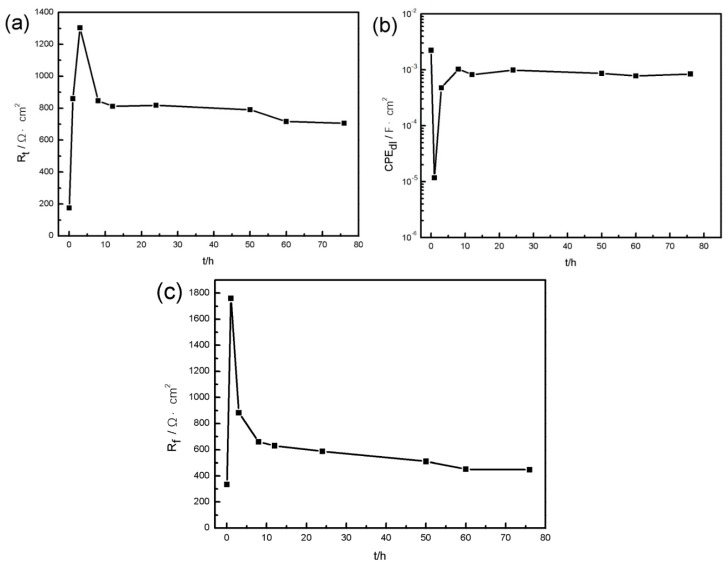
Fitting results of resistances and constant phase element during the immersion test: (**a**) charge transfer resistance *R*_t_, (**b**) constant phase element CPE_dl_, (**c**) film resistance *R*_f_.

Figure 5 shows the EIS fitting results including *R*_t_ (Figure 5a), CPE_dl_ (Figure 5b), and *R*_f_ (Figure 5c). It can be found that *R*_t_ of the specimen first increases with immersion time, but after immersion for 3 h, the value of *R*_t_ begins to decrease and maintains a lower level, simultaneously, *R*_f_ shows the same variation tendency. However, CPE_dl_ represents an opposite performance. It indicates that an effective barrier appeared during the initial stage of corrosion, then the protective film was broken and localized corrosion developed, coinciding with the OCP result.

#### 3.1.3. Hydrogen Collection

**Figure 6:**
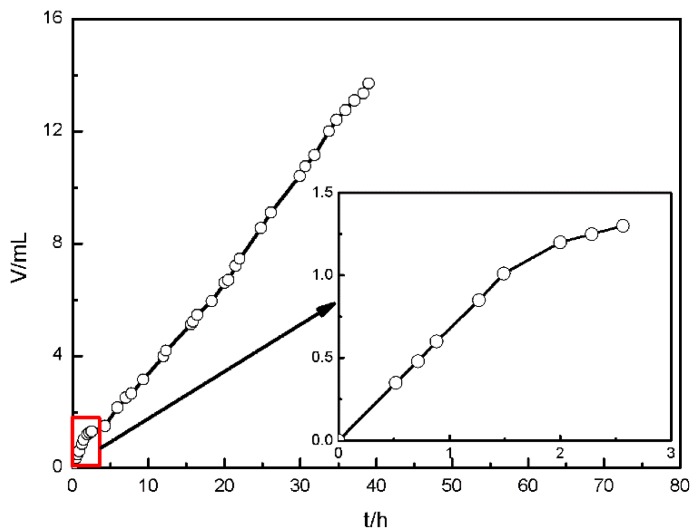
Volume of hydrogen evolution of AZ91D in simulated HA solution.

The corrosion rate of sample can be evaluated by the volume of the hydrogen collected in situ [32]. Figure 6 shows the volume of hydrogen generated by AZ91D immersed in simulated solution. In the first 1.5 h, the volume of hydrogen has a rapid growth, suggesting that the corrosion rate was fast. During 1.5–2.5 h, the volume increases slowly, indicating that the corrosion rate slowed down. After that, the volume of hydrogen extends a certain value per unit time, implying that the corrosion rate kept stable. This is in good agreement with the results obtained from OCP and EIS measurements.

#### 3.1.4. Surface Appearance of AZ91D Immersed for Various Time

In order to have a further investigation on the corrosion process, samples were immersed in simulated HA solution for different time (1.5, 3, 8, 48 h). After immersion, the surface product and morphology were observed through SEM equipped with EDX and CLSM.

**Figure 7:**
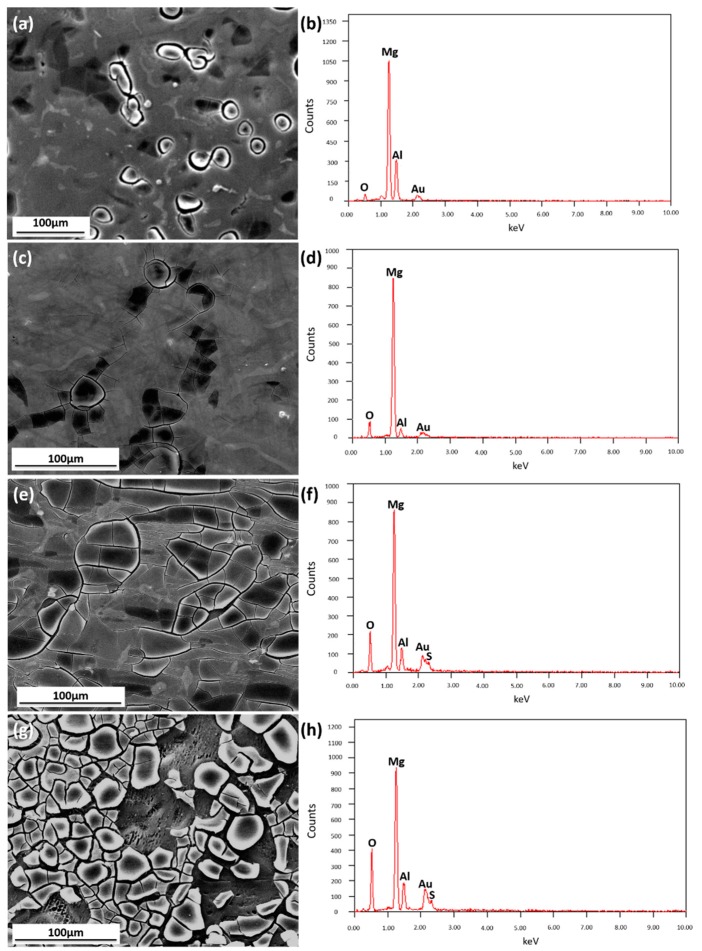
SEM (**a**,**c**,**e**,**g**) images and EDX results of samples immersed in solution for 1.5 h (**a**,**b**), 3 h (**c**,**d**), 8 h (**e**,**f**) and 48 h (**g**,**h**).

Figure 7 displays the SEM surface morphology and the corresponding EDX of AZ91D immersed in simulated HA solution for 1.5, 3, 8 and 48 h. In Figure 7a, there is a few small pitting points, and the surface is almost flat, even β-phase can be recognized on the surface. Figure 7b indicates a low O/Mg ratio. As time increases, small pitting points grow up and product film forms on the surface. Figure 7c shows that the product film ruptures and localized corrosion begins to deteriorate, and the signal ratio of O/Mg increases. After 8 h of immersion, the surface is seriously damaged (Figure 7e), and the signal peak of S appears evidently (Figure 7f). Figure 7g suggests that AZ91D surface is terribly damaged after being immersed for 48 h. The EDX measurement shows that the signal ratio of O/Mg increases over time, and Al content is almost unchanged. That is likely because the grains have corroded away, leaving a fine network structure consisting of higher aluminum content.

**Figure 8:**
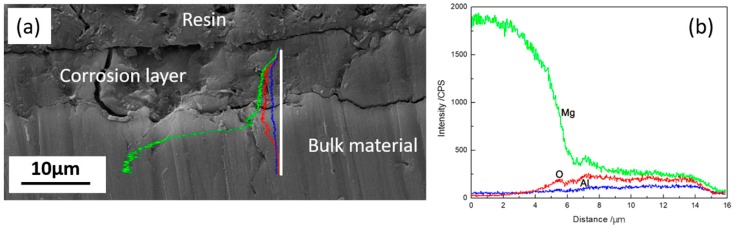
The cross-section of sample immersed in HA solution for 48 h (**a**) and the corresponding linear EDX spectra (**b**).

Figure 8 shows the cross-section of corrosion layer formed on AZ91D alloy after immersion for 48 h. It reveals a discontinuous and cracked corrosion film of about 10 μm, and O enrichment is found in the corrosion layer, meanwhile Al has nearly the same content between the bulk material and corrosion layer, which is in good agreement with Figure 7.

**Figure 9:**
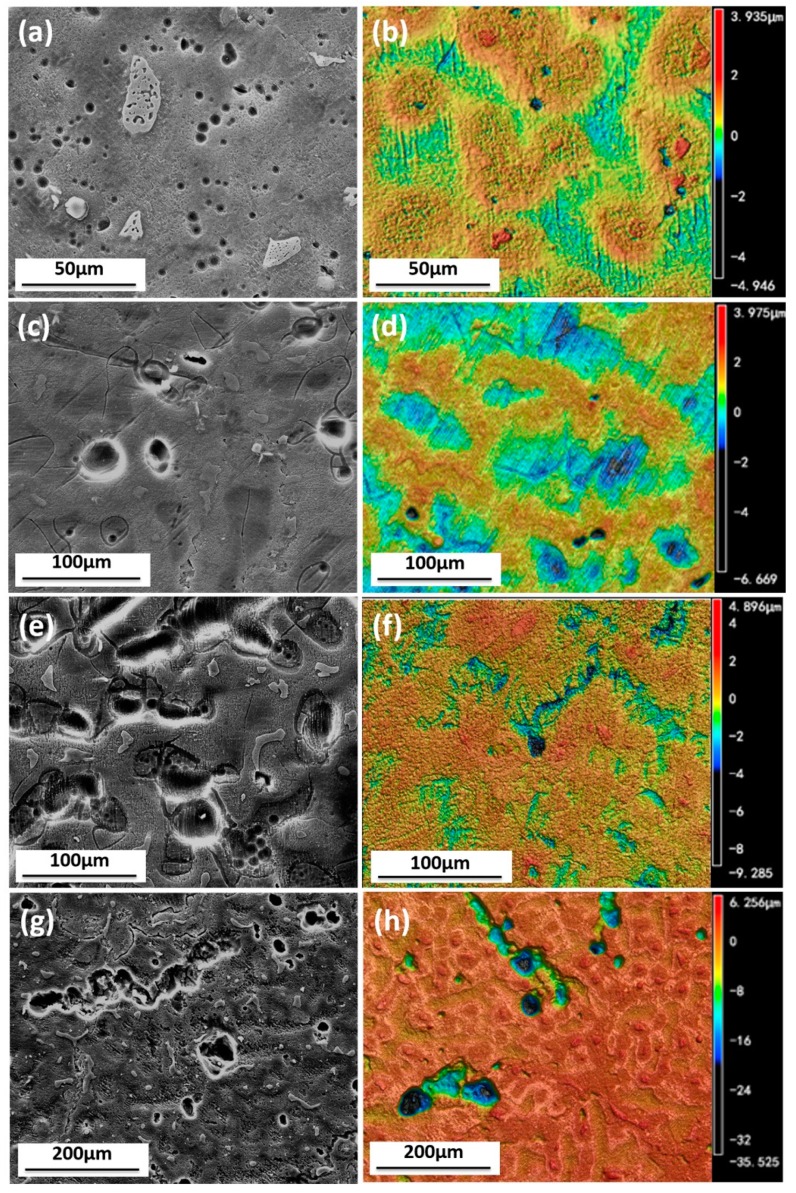
SEM (**a**,**c**,**e**,**g**) and CLSM (**b**,**d**,**f**,**h**) images of samples immersed in solution for 1.5 h (**a**,**b**), 3 h (**c**,**d**), 8 h (**e**,**f**) and 48 h (**g**,**h**) after rust removal.

Figure 9 shows the surface morphology of samples immersed for different time, after rust removal in 1000 mL deionized water containing 200 g CrO_3_, 10 g AgNO_3_, and 20 g Ba(NO_3_)_2_. At the first 1.5 h, the pit depth on the AZ91D magnesium alloy surface in the medium was about 5 μm, with a surface diameter of less than 3 μm. It depicts that the corrosion was initiated from pitting corrosion. Both pit depth and surface diameter on specimen surface tended to grow with the increase of immersion time. These images reveal that small pits united and therefore big pits formed finally.

### 3.2. The Influence of Ions

#### 3.2.1. Surface Appearance of AZ91D Immersed in Various Solutions

Simulated HA solution contains four kinds of water-soluble ions, to explore the influence of each ion towards AZ91D, immersion test was carried out. Samples were immersed in five types of solutions for 11 days. The original composition of simulated HA solution is mentioned in Section 2.1, and the four other solutions were respectively lack of ${SO}_{4}^{2-}$, ${NO}_{3}^{-}$, ${NH}_{4}^{+}$ and Cl^−^.

**Figure 10:**
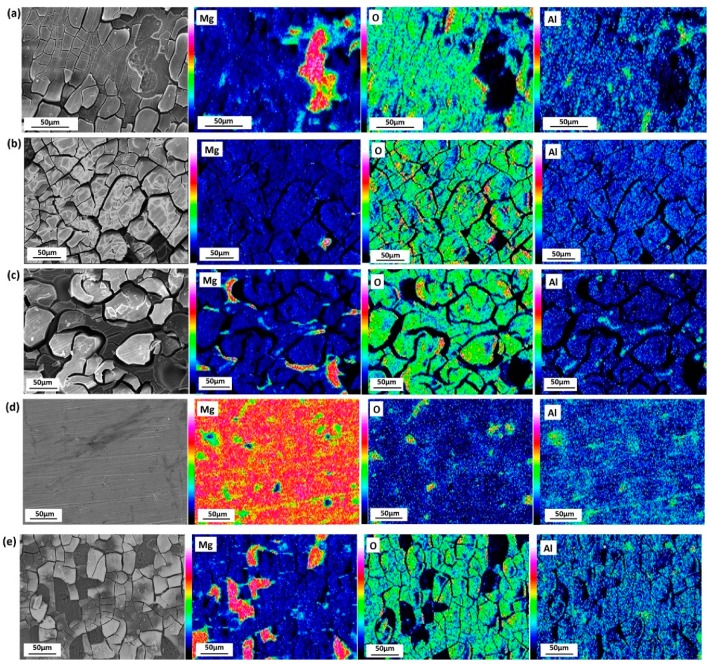
SEM images and corresponding X-ray maps of Mg, Al and O for AZ91D after immersed in simulated HA solution (**a**), and solutions at the shortage of ${SO}_{4}^{2-}$ (**b**), ${NO}_{3}^{-}$ (**c**), ${NH}_{4}^{+}$ (**d**), Cl^−^ (**e**) for 11 days.

The SEM micrographs and corresponding EDX maps of the surface of the tested samples are shown in Figure 10. After 11 days immersion, the product film ruptures terribly, even slips off the bulk, and the bare Mg matrix is exposed, as shown in Figure 10a. As Figure 10b discloses, when lacking ${SO}_{4}^{2-}$, the film is broken evenly, but barely comes off the matrix. As can be seen from Figure 10c, when ${NO}_{3}^{-}$ is absent, localized corrosion is severe, Al enrichment is found in the remaining bulk. Figure 10d shows that the surface of sample is damaged slightly when short of ${NH}_{4}^{+}$. As Figure 10e reveals, Cl^−^ does not show much influence towards corrosion, because the surface appearance is like that shown in Figure 10a.

**Figure 11:**
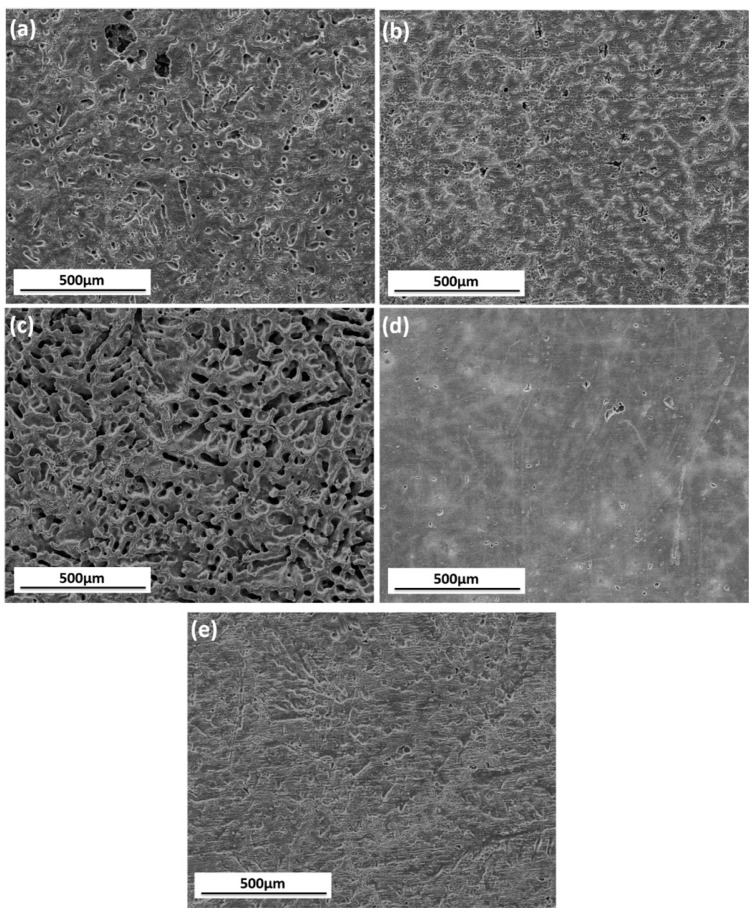
SEM images of AZ91D immersed in simulated HA solution (**a**), and solutions at the shortage of ${SO}_{4}^{2-}$ (**b**), ${NO}_{3}^{-}$ (**c**), ${NH}_{4}^{+}$ (**d**), Cl^−^ (**e**) for 11 days after rust removal.

Figure 11 shows the surface morphology of tested samples after rust removal. Figure 11a shows that overall corrosion and localized corrosion develop on the surface when immersed in simulated HA solution. In the absence of ${SO}_{4}^{2-}$, less serious localized corrosion pits are formed on the surface, as Figure 11b shows. The morphology in Figure 11c reveals the terrible corroded surface resulting from the lack of ${NO}_{3}^{-}$, and the corrosion pits are distributed around the resistant β-phase network, suggesting that ${NO}_{3}^{-}$ holds back the corrosion process as it does for some other metals [33]. The slight pitting corrosion shown in Figure 11d discloses that the absence of ${NH}_{4}^{+}$ slows down the corrosion process drastically. The shortage of Cl^−^ alleviates the pitting corrosion of AZ91D (Figure 11e), indicating that Cl^−^ helps to develop localized corrosion. The pre-study shows that Cl^−^ promotes rapid attack in neutral aqueous solutions and acidic solutions [34,35].

#### 3.2.2. Electrochemical Measurements

In order to obtain further information about the influence of ions, electrochemical measurements were carried out. The original composition of simulated HA solution is mentioned in Section 2.1. When it comes to the influence ${SO}_{4}^{2-}$, the concentration of ${SO}_{4}^{2-}$ is adjusted from 0.05 to 0, 0.01, 0.05, 0.1 M, respectively. This is consistent with the rest of the three ions.

**Figure 12:**
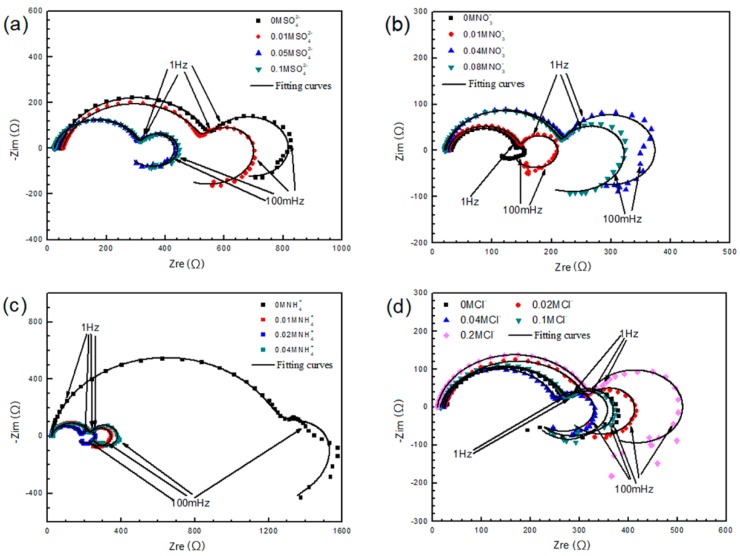
EIS dependence on ${SO}_{4}^{2-}$ (**a**), ${NO}_{3}^{-}$ (**b**), ${NH}_{4}^{+}$ (**c**), Cl^−^ (**d**) concentration of AZ91D.

Figure 12 shows the Nyquist diagrams of AZ91D samples immersed in solutions containing different concentrations of water-soluble ions, exhibiting various electrochemical characteristics. The equivalent circuit is Model A in Figure 4a. The shape of the complex diagram changes with ions concentration and displays how the ions influenced the corrosion process. Figure 12a indicates that ${SO}_{4}^{2-}$ is harmful to AZ91D, but the impedance arcs reach a stable state once the concentration of ${SO}_{4}^{2-}$ exceeds a certain value. Figure 12b reveals that ${NO}_{3}^{-}$ is a kind of protective ions, as the impedance modulus value tends to grow large with the increasing concentration. Figure 12c shows that ${NH}_{4}^{+}$ is the most significant ion in this study, for the impedance modulus value decreases by more than four times with only 0.01M ${NH}_{4}^{+}$. Figure 12d suggests that Cl^−^ does not show evident influence. Therefore, ${NO}_{3}^{-}$ and ${NH}_{4}^{+}$ are the two most obvious ions among the four kinds of water-soluble ions.

**Figure 13:**
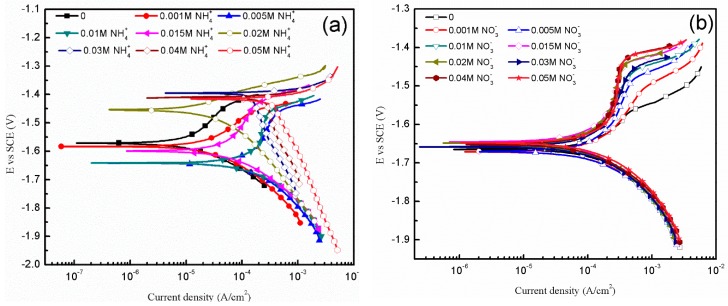
Polarization curves of AZ91D in solutions of different ${NH}_{4}^{+}$ (**a**) and ${NO}_{3}^{-}$ (**b**) concentration.

To know more about the influence of ${NO}_{3}^{-}$ and ${NH}_{4}^{+}$ towards specimens, further efforts were made through polarization curves, as shown in Figure 13. The corrosion potentials (*E*_corr_), corrosion current densities (*i*_corr_) and corrosion rate (*r*_c_) values of polarization curves in Figure 13 are summarized in Table 3. *E*_corr_, *i*_corr_ of the samples are obtained by the Tafel extrapolation method; *r*_c_ (mm/a) is related to *i*_corr_ (mA/cm) [36] using

(1) $$r_{c}=22.85i_{corr}$$

**Figure 14:**
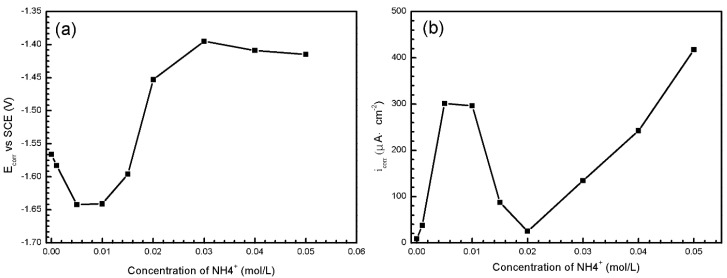
The *E*_corr_ (**a**) and *i*_corr_ (**b**) values of samples in Figure 13a.

Figure 13a reveals the anodic and cathodic polarization behaviors of the specimens exposed to solutions containing ${NH}_{4}^{+}$ of 0 to 0.05 M. As the acquired curves reveal evident curvature, the Tafel extrapolation of the anodic and cathodic branches are performed using the Tafel Fit in the P4000+. The identified dynamic polarized *E*_corr_ and *i*_corr_ obtained from Figure 13a are summarized in Figure 14. The values of *i*_corr_ show a drastic change when the concentration of ${NH}_{4}^{+}$ reaches 0.005 M. The decrease of *i*_corr_ in solution of 0.02 M ${NH}_{4}^{+}$ is likely due to the formation of corrosion product. The cathodic branch shows an extensive linear Tafel region and the cathodic current density is obviously promoted with the increasing concentration of ${NH}_{4}^{+}$. In addition, the slope of anodic branch has a tendency to decrease with the increasing concentration of ${NH}_{4}^{+}$, so it indicates that the breakdown of product coverage on corrosion surface increases the active region of corrosion reaction [37]. It can be inferred that ${NH}_{4}^{+}$ hinders the formation of product hydroxide/oxide film by the consuming OH^−^. As shown in Table 3, the corrosion rate of AZ91D increases nearly tenfold from 0.8598 mm/a to 6.882 mm/a when ${NH}_{4}^{+}$ changes from 0.001 to 0.005 M. When ${NH}_{4}^{+}$ reaches 0.02 M, the corrosion rate drops down due to the productive film. However, as ${NH}_{4}^{+}$ concentration increases, the corrosion rate increases linearly. Former study indicates that the corrosion rate of AZ91D immersed in 1 M NaCl solution is 0.91 mm/a [36], close to the corrosion rate of AZ91D immersed in simulated HA solution with 0.001 M ${NH}_{4}^{+}$. It indicates that ${NH}_{4}^{+}$ is largely responsible for the terrible corrosion behavior in simulated HA solution.

Figure 13b shows the potentiodynamic polarization curves of samples immersed in solutions containing ${NO}_{3}^{-}$ of 0 to 0.05 M. There is little difference among cathodic Tafel slopes, reflecting similar electrochemical reactions of hydrogen evolution. Table 3 shows that the values of *E*_corr_, *i*_corr_ and *r*_c_ fluctuate slightly with the change of ${NO}_{3}^{-}$ concentration. However, Figure 11c reveals that ${NO}_{3}^{-}$ does have an important influence on the corrosion behavior. It can be seen from Figure 13b that with the increasing concentration of ${NO}_{3}^{-}$, the slope of anodic branch increases, indicating more passive product film coverage on the corrosion surface. Therefore, the protective role ${NO}_{3}^{-}$ plays can be attributed to the formation of passive product film.

#### 3.2.3. XRD Analysis

**Figure 15:**
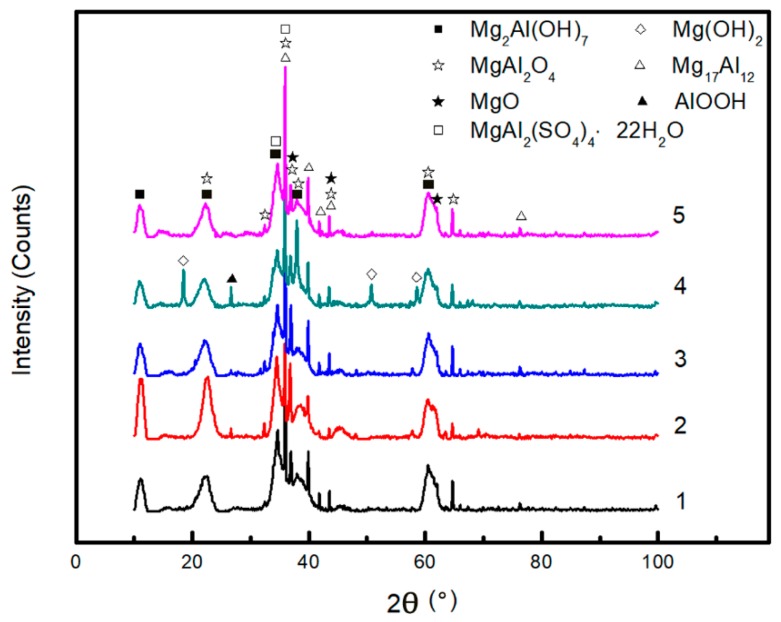
XRD patterns for the product of AZ91D after immersed in simulated HA solution (**1**), and at the shortage of ${SO}_{4}^{2-}$ (**2**), ${NO}_{3}^{-}$ (**3**), ${NH}_{4}^{+}$ (**4**), Cl^−^ (**5**) for 11 days.

After the immersion test, the corrosion product slipping off was collected and powdered to be analyzed through XRD. As shown in Figure 15, MgO and MgAl_2_O_4_ are the main corrosion products. As shown in No.2 XRD pattern, the absence of ${SO}_{4}^{2-}$ results in an evidently lower MgAl_2_(SO_4_)_4_·22H_2_O peak, indicating that MgAl_2_(SO_4_)_4_·22H_2_O is also an important product. It reveals from the No.4 XRD pattern that the product collected in solution lack of ${NH}_{4}^{+}$ contained Mg(OH)_2_, which barely exists in other results, identifying ${NH}_{4}^{+}$ accelerates corrosion by consuming OH^−^ in a straightforward way.

## 4. Discussion

### 4.1. The Corrosion Behavior of AZ91D Alloy in the Simulated HA Solution

As described earlier, the corrosion rate of AZ91D immersed in simulated HA solution changed with time. Figure 2, Figure 3 and Figure 6 indicate that the corrosion process firstly suppressed then accelerated and finally reached stable. The corrosion resistance reached peak value after about 3 h immersion due to the formation of passive film on the *α*-matrix surface. ${NO}_{3}^{-}$ played an important role in the process, according to Figure 11c and Figure 13b. In the meantime, the Cl^−^ preferentially attacked the weak sites of surface film to form the active sites [38]. Then the localized corrosion initiated at the active sites, as depicted in Figure 9a.

When the immersion time exceeded 3 h, the corrosion rate increased. As can be seen from Figure 7c, the tiny cracks undermined the alloy matrix, and bare Mg was exposed to the solution, causing the reduction of corrosion resistance. When the cracks were so large that the surface could not hold corrosion product, part of the film began to slip. Therefore, barer matrix was exposed and corrosion became serious. According to the mechanism proposed by Chen [30], the aluminum concentration in the solution increased due to the dissolution of the eutectics (α-Mg + β-Mg_17_Al_12_), once aluminum concentration achieved a certain value, the precipitation of MgAl_2_(SO_4_)_4_·22H_2_O was formed as reaction (2), which occurred mainly in the pitting area. Figure 11b suggests that the corrosion pits still existed, but they were much more alleviative when ${SO}_{4}^{2-}$ was absent in the solution. This indicates that Cl^−^ gave rise to the localized corrosion and ${SO}_{4}^{2-}$ aggravated corrosion pits.

(2) $${\mathrm{Mg}}^{2+}+2{\mathrm{Al}}^{3+}+4{SO}_{4}{}^{2-}+22H_{2}O↔{\mathrm{Mg}\mathrm{Al}}_{2}{\left({SO}_{4}\right)}_{4}\cdot 22H_{2}O$$

The main corrosion products of AZ91D immersed in simulated HA solution for 11 days were MgO, MgAl_2_O_4_ and MgAl_2_(SO_4_)_4_·22H_2_O, as shown in Figure 15. The reason why Mg(OH)_2_ was absent was that ${NH}_{4}^{+}$ consumed OH^−^, therefore Mg^2+^ could not combine with OH^−^ to form protective Mg(OH)_2_ film. Without the protection of Mg(OH)_2_, the corrosion was more serious, which could be proved by Figure 11d and Figure 12c. It should be noted that with the development of corrosion, ${NH}_{4}^{+}$ ran out around the localized corrosion pits, the resistant β-phase began to react with OH^−^, leading to the formation of Mg_2_Al(OH)_7_ and AlOOH.

### 4.2. The Corrosion Mechanism of AZ91D in the Simulated HA Solution

**Figure 16:**
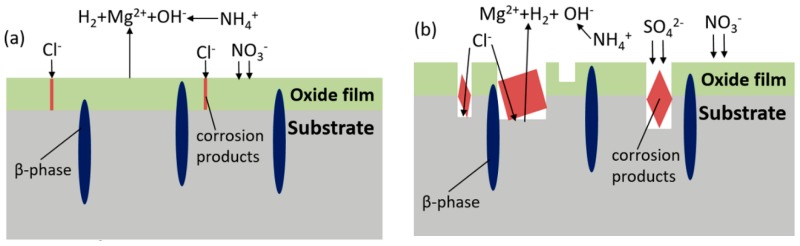
Corrosion process diagram of AZ91D consisting of a first stage (**a**) and a second stage (**b**) immersed in HA solution.

Taking into account both the corrosion process of AZ91D in the simulated HA solution and the influence of ions, a schematic diagram consisting of two stages is proposed (Figure 16) in order to explain the corrosion mechanism of AZ91D in simulated HA solution.

- First stage (shown in Figure 16a). According to Song [17], the anodic reaction becomes the following equations:
  (3a) $$\mathrm{Mg}\;\to {\;\mathrm{Mg}}^{+}+e^{-}$$
  (3b) $${\mathrm{Mg}}^{+}\to {\;\mathrm{Mg}}^{2+}+e^{-}$$
  The overall reaction:
  (3) $$\mathrm{Mg}\;\to {\;\mathrm{Mg}}^{2+}+2e^{-}$$
  The cathodic reaction:
  (4) $$2H_{2}O+2e^{-}\;\to {\;H}_{2}+2{OH}^{-}$$
  Mg(OH)_2_ is supposed to be formed on the surface, but the process is thwarted by ${NH}_{4}^{+}$:
  (5) $${NH}_{4}^{+}+\;{OH}^{-}\;\to \;{NH}_{3}\cdot H_{2}O$$
  Therefore, the product film is formed once the sample is exposed to the solution, and the main components refer to MgO instead of Mg(OH)_2_. The existence of ${NO}_{3}^{-}$ helps to form a passive film on the surface, so the corrosion rate is soon limited. Cl^−^ brings about the localized corrosion, therefore tiny corrosion pits appear where the sites are active.
- Second stage (shown in Figure 16b). As the corrosion develops, the resistant β-Mg_17_Al_12_ dissolves, thus aluminum concentration elevates and the precipitation of MgAl_2_(SO_4_)_4_·22H_2_O is formed according to Reaction (2), resulting in a higher corrosion rate. Corrosion continues to develop in pits, where bare α-matrix is firstly to be corroded and ${NH}_{4}^{+}$ is consumed up by OH^−^, then β-phase reacts with OH^−^, generating Mg_2_Al(OH)_7_ and AlOOH. Cracks generally appear on the coverage and film drops out, even in a severe process, Mg particle surrounded by completely corroded material falls into solution, resulting in bare Mg exposed to the solution. Meanwhile, ${SO}_{4}^{2-}$ and Cl^−^ keep attacking to form deeper pits.

## 5. Conclusions

The corrosion process and influence of ions of AZ91D samples in simulated HA solution were studied. The corrosion process was monitored by morphology observation, electrochemical experiment and hydrogen collection, and corrosion products were analyzed using EDX. The influence of four main ions in haze was examined by potentiodynamic polarization curves, SEM equipped with EDX, and the corrosion products were confirmed by XRD. The results can be summarized as follows:

- ${SO}_{4}^{2-}$, ${NO}_{3}^{-}$, ${NH}_{4}^{+}$, Cl^−^ are the four main water-soluble ions in simulated HA solution. ${SO}_{4}^{2-}$ and Cl^−^ are aggressive towards AZ91D, causing and aggravating pitting corrosion. The absorption of ${NO}_{3}^{-}$ prevents samples, especially α-matrix from severe corrosion by generating passive film on the surface. The combination of ${NH}_{4}^{+}$ and OH^−^ blocks the formation of Mg(OH)_2_, therefore corrosion process accelerates drastically.
- The corrosion attack of AZ91D immersed in simulated HA solution mainly takes place in α-phase matrix. Pitting corrosion is the main damage taking place on the surface. In addition, shallow pits resulting from Cl^−^ are superimposed to form deep pits due to ${SO}_{4}^{2-}$, and Mg particle undermining might take place. The main corrosion products are MgO, MgAl_2_O_4_ and MgAl_2_(SO_4_)_4_·22H_2_O.
- With the development of the corrosion, α-matrix and β-Mg_17_Al_12_ are dissolved, and localized corrosion aggravates, so the corrosion rate rises and finally stabilizes.

## Figures and Tables

**Table 1:** Chemical composition of AZ91D (in wt.%).

Al	Zn	Mn	Si	Ni	Fe	Cu	Mg
8.56	0.54	0.22	0.054	<0.005	<0.005	<0.005	Bal.

**Table 2:** The element composition of regions 1 and 2 in Figure 1 (in wt.%).

Region	Mg	Al	Zn
1	97.72	1.56	–
2	70.21	27.25	1.86

**Table 3:** Tafel extrapolation evaluated *E*_corr_, *i*_corr_ and *r*_c_ values of AZ91D at solutions of different ${NH}_{4}^{+}$ and ${NO}_{3}^{-}$ concentration.

Concentration (mol/L)	${NH}_{4}^{+}$			${NO}_{3}^{-}$		
	*E*_corr_ (V vs. SCE)	*i*_corr_ (μA/cm^2^)	*r*_c_ (mm/a)	*E*_corr_ (V vs. SCE)	*i*_corr_ (μA/cm^2^)	*r*_c_ (mm/a)
0	−1.566	8.68	0.1983	−1.665	246.7	5.637
0.001	−1.583	37.63	0.8598	−1.670	224.0	5.118
0.005	−1.642	301.2	6.882	−1.670	265.2	6.060
0.01	−1.641	296.2	6.768	−1.653	172.7	3.946
0.015	−1.596	87.52	2.000	−1.645	203.0	4.639
0.02	−1.453	25.07	0.5729	−1.648	239.7	5.477
0.03	−1.395	134.5	3.073	−1.657	268.5	6.135
0.04	−1.409	242.3	5.537	−1.652	244.1	5.578
0.05	−1.415	417.6	9.542	−1.648	280.2	6.403
